# Mapping physical activity patterns in hospitalised patients with moderate to severe acquired brain injury - MAP-ABI: Protocol for an observational study

**DOI:** 10.1016/j.heliyon.2023.e21927

**Published:** 2023-11-08

**Authors:** Vibeke Wagner, Pi Gravesen, Emma Ghaziani, Markus Harboe Olsen, Christian Gunge Riberholt

**Affiliations:** a Department of Brain and Spinal Cord Injury, The Neuroscience Centre, Copenhagen University Hospital – Rigshospitalet, Copenhagen, Denmark; b Department of Neuroanaesthesiology, The Neuroscience Centre, Copenhagen University Hospital – Rigshospitalet, Copenhagen, Denmark

**Keywords:** Accelerometry, Brain injuries, Inpatients, Mobility limitation, Neurological rehabilitation, Observational study, Physical activity, Physical inactivity, Sedentary behaviour

## Abstract

**Introduction:**

The physical activity level in patients hospitalised for rehabilitation across multiple diagnoses is low. Moderate to severe acquired brain injury further reduces activity levels as impaired physical and cognitive functioning affect mobility independence. Therefore, supervised out-of-bed mobilisation and physical activity training are essential rehabilitation strategies. Few studies have measured the physical activity patterns in the early phases of rehabilitation after moderate to severe brain injury.

**Objectives:**

To map and quantify physical activity patterns in patients admitted to brain injury rehabilitation. Further, to investigate which factors are associated with activity and if the early physical activity level is associated with functional outcome at discharge.

**Methods:**

This observational study includes patients admitted to rehabilitation after moderate to severe acquired brain injury. Mobility and physical activity patterns are measured continuously during rehabilitation at two separate seven-day periods using a wearable activity tracker. Activity will be categorised into four levels and presented descriptively. Linear and logistic regression models will analyse associations between descriptive variables and activity levels.

**Discussion:**

This protocol describes an observational study investigating patients' mobility and physical activity patterns with moderate to severe acquired brain injury during in-hospital rehabilitation. The ability to increase the amount of mobilisation and physical activity in subgroups may have profound consequences on the rehabilitation outcome. Furthermore, data from this study may be used to inform a large variety of trials investigating physical rehabilitation interventions. (NCT05571462).

## 1 Introduction

Physical activity is an essential prerequisite for general health and well-being and is recommended for all ages and health conditions. Physically active individuals have a decreased risk of degenerating neurological diseases, cancer, and diabetes [1].

Regardless of age, hospitalised patients with acquired brain injury have large daily proportions of inactive periods in both the acute and the subacute phases of rehabilitation [[2], [3], [4], [5], [6]]. Studies have shown that people with traumatic brain injury, brain haemorrhages or stroke are physically inactive between 77 % and 96 % of the day [[2], [3], [4], [5]], and 34 %–74 % of the daytime is spent socially idle and alone [[3], [4], [5]]. Physical activity is highly dependent on the severity of brain injury, as demonstrated by Hassett et al. [5] and Kjeldsen et al. [4]. The physiological consequences of immobilisation are well-documented in both the healthy population and in people with other diagnoses than neurological [7,8].

A large part of the improvement in functioning after an acquired brain injury occurs within the first three months when patients are typically hospitalised. A positive dose-response effect of motor re-learning is accepted within neurorehabilitation, and it is explained by learning theories and the neuroplastic characteristics of the central nervous system [9,10]. However, the severity of the brain injury affects physical and cognitive functioning, including the ability to mobilise independently with or without helping aids. Furthermore, the patients may depend on assistance in multiple other functions and activities of daily living due to unstable vital functions or impaired level of consciousness. For instance, low arousal will markedly affect the physical activity level and require several resting periods throughout the day [2,4]. Furthermore, the ability to be physically active may depend on secondary complications (e.g., fractures or spasticity), staff resources, daily planning, or access to appropriate rehabilitation aids. Therefore, severely injured patients depend on staff to lift them out of bed to a chair or assist in mobility activities like transfers and walking [11]. One of the early rehabilitation goals is to increase mobility independence by increasing the physical activity level through structured exercise or more passive mobilisation strategies [11,12].

### 1.1 Physical activity

According to the World Health Organization (WHO), “physical activity” is defined as *“bodily movement that requires energy expenditure produced by skeletal muscles”* However, physical activity is also used in general terms, e.g., when describing the overall “physical activity level” for an individual or a group. The term “physical activity” can also be used for more specific exercises to increase the general activity level, like going for a run or doing 30 min of fitness training [1]. The International Classification of Functioning, Disability, and Health (ICF) defines mobility as *“moving by changing body position or location or by transferring from one place to another, by carrying, moving or manipulating objects, by walking, running or climbing, and by using various forms of transportation”* [13]. Patients with neurological impairments are less able to be physically active independently. Therefore, in this study, physical activity can be movement without energy expenditure, e.g. when patients are mobilised by staff.

### 1.2 Physical activity monitoring

A valid and well-documented method for quantifying physical activity is accelerometers embedded in wearable devices with or without user feedback [14]. The effect of wearable activity devices with feedback has been widely investigated and shows significant changes in physical activity levels when measured by step count [14,15]. Thereby, such devices have the potential to directly motivate the user (i.e. the patient) and healthcare personnel and carers [15]. However, for an observational study, feedback is not necessary. The *SENS motion®* (SENS Innovation ApS, Copenhagen, Denmark) activity tracker is developed for research purposes or healthcare interventions [16,17].

### 1.3 Study objectives and hypothesis

The primary objective of this study is to characterise mobility and physical activity levels in patients with moderate to severe acquired brain injury using four prespecified physical activity levels (*physically active, physically sedentary, physically inactive, and sleep)* at the early and late phases of hospital rehabilitation. Secondly, we will investigate which factors are associated with a higher physical activity level at the early and late phases of admission. Lastly, we will investigate whether the level of physical activity at the early admission phase is associated with better functional outcomes at discharge.

We hypothesise that patients are less mobilised and physically active in the early phase of hospital rehabilitation and that their physical activity level increases before discharge. Furthermore, we expect an association between change in physical activity levels and overall physical function over time, while physical and cognitive factors might act as modifiers or confounders.

## 2 Methods

### 2.1 Study design

This is an observational study that examines physical activity patterns in a cohort of patients admitted for sub-acute rehabilitation. The study takes place at one of two highly specialised hospital departments for brain injury rehabilitation in Denmark. The department offers multidisciplinary rehabilitation to the most complex cases of moderate to severe acquired brain injury of traumatic and non-traumatic aetiologies. The rehabilitation programme follows Danish guidelines [18,19] and evidence based recommendations for brain injury rehabilitation [20]. The rehabilitation department has 18 beds for patients ≥18 years of age. In most cases, the length of stay is between 6 and 14 weeks, and patients are discharged when medical care is no longer needed (e.g., tracheal tube) or when the neuropsychological and physical impairments (e.g., severe agitation and muscular hypertonia) can be managed in less specialised facilities. After discharge, further rehabilitation is offered in the patient's municipality or regional hospitals with neurological rehabilitation departments.

Study participants will receive standard care and rehabilitation throughout the study. Standard care consists of a 24-h interdisciplinary team approach around individual patient-centred goals. Up to 7 professions are in the team, depending on the patient's needs. Management of secondary complications and early out-of-bed mobility are core rehabilitation practices. The purpose is to stimulate arousal, awareness, and neuromuscular functions to facilitate physical independence. Other early focuses are facial and oral functions and participation in personal activities. Consciousness, cognitive and communicative abilities are continuously assessed and incorporated into the rehabilitation activities.

The study will run from November 2022 until 100 patients are included. Reporting will adhere to the STROBE statement [21,22].

#### 2.1.1 Participants

All patients admitted to the brain injury rehabilitation department are screened for eligibility. Patients ≥18 years of age are eligible to participate. Study enrolment takes place within the first three days of admission. The primary investigator and research assistants are responsible for the recruitment and collection of informed consent. Most patients are at admission unable to give informed consent, and proxy consent from the nearest relative and consent from the study guardian will be obtained. Patient consent will be obtained if patients regain the ability to consent during the study. All included patients must have an expected hospital stay of more than two weeks in the department. Patients with fractures of the pelvis or spine and those who have undergone amputation of the lower extremities are not eligible, as physical activity can be contraindicated.

### 2.2 Procedures

Physical activity data will be collected for two seven-day periods during hospitalisation. Period one (P1) starts 1–3 days after admission and enrolment, and Period two (P2) starts 7–10 days before discharge.

The *SENS motion®* is a water-proof body-worn activity tracker placed on the leg and the upper body to measure physical activity and body posture from accelerometer-driven data. The *SENS motion®* samples at 12 Hz via a triaxial accelerometer. The recorded raw data are automatically transmitted to a secure web server whenever sensors are within reach of the *SENS motion®* app installed on a tablet or phone. The *SENS motion®* algorithms are developed based on a single sensor system. However, an algorithm based on data from two sensors can distinguish between lying, sitting, and standing and if the patient is in motion. This enables us to categorise activity data into active and sedentary positions [16].

In this study, one activity tracker is placed on the thigh approximately 10 cm above the lateral epicondyle on the non-paretic side or the side with the most activity. The other sensor is placed on the torso, either on the sternum or on the back, depending on the patient's preference or as judged most considerate by the research assistant. Two to four clinicians (therapists or nurses) will be responsible for the activity monitors, i.e., distribution, placement, daily control, and disinfection after use. Standard care and rehabilitation continue according to the patient's rehabilitation plan when the sensors are activated. During and after P1 and P2, the patient's rehabilitation team will secure that sensor is not coming off and record how the sensor is tolerated. After P1 and P2, the research assistants are responsible for cleaning each sensor, transferring data, and turning the sensor off. *SENS motion®* has been found reliable and valid in one study examining the activity level of patients admitted with knee osteoarthritis [23] and another examining slow and fast-walking hospitalised patients [17]. The sensors are attached with adhesive tape, which in rare cases, can trigger a local allergic reaction. Should this occur, the monitor is removed immediately, and no more data will be recorded.

#### 2.2.1 Data collection

*Baseline characteristics:* We will extract the following data from the electronic patient chart, including sex, age, primary diagnosis, comorbidities, body mass index (BMI), injury date and time to study inclusion, and length of stay in the intensive care unit (ICU). We report the following scores to describe the severity of the brain injury and functioning at admission (baseline) to the rehabilitation department: The Full Outline of UnResponsiveness (FOUR) score [24]; the Early Functional Abilities Scale (EFA) [25]: total score and sensory-motor subscale scores (item 9–15); the Functional Independence Measure (FIM) [26]: total score, motor subscale (FIM-Motor), and cognitive subscale (FIM-Cog); and the Coma Recovery Scale-Revised (CRS-R) [27] (Table 1).

**Table 1:** List of variables.

	Exposure	Predictor	Confounder	Modifier	Outcome
Exposure variable					
Level of physical activity during P1	X				X
Level of physical activity during P2	X				X
**Baseline variables (descriptive)**					
Sex		X	X	X	
Age		X	X	X	
Primary diagnoses			X	X	
Comorbidities		X	X	X	
BMI (admission)		X	X	X	
Injury date and time to study inclusion					
Length of stay in ICU		X	X	X	
FOUR score		X			
Total EFA score and sensory-motor subscale (items 9–15)		X	X	X	
Total FIM score, FIM-Motor and FIM-Cognitive subscales		X	X	X	
CRS-R		X	X	X	
**Other variables (possible confounders or modifiers)**					
Number of complications during P1 and P2 (yes/no; counts; date)			X	X	
- Tracheal tube and decannulation					
- Craniectomy and replacement					
- Constant monitoring of vital parameters					
- Constant connection to oxygen or medications					
- Infections and duration					
- Gastric tube operation					
- Urinary or anal catheter					
- Contractures of lower limbs*					
- Syncope during upright positions					
- Visual deficits*					
- Nausea or dizziness during therapy/out of bed					
- Agitated behaviour (ABS>22)					
Length of stay at the rehabilitation unit		X	X	X	
**Outcome variables (descriptive), discharge**					
FIM-motor sub-score					X
FIM-Cognitive sub-score					X
30-s sit-to-stand test				X	X
Functional Ambulation Category				X	X
10-m walk test				X	X
6-min walk test				X	X

**P1:** Period 1 (early phase admission); **P2:** Period 2 (late phase admission); **BMI**: Body Mass Index; **ICU**: Intensive care unit; **FOUR**: Full Outline of UnResponsiveness; **EFA**: Early Functional Abilities Scale total score; **FIM**: Functional Independence Measure; **CRS-R**: Coma Recovery Scale-Revised; **ABS:** Agitated Behaviour Scale. *We record contractures and visual deficits confirmed to influence the physical activity level of the patient.

*Level of physical activity (exposure variable):* We will analyse and classify the level of physical activity during P1 and P2 into four categories: *physically active, physically sedentary, physically inactive, and sleeping*. The raw data enables us to define several activity categories using the abovementioned algorithm. The sensors register even small movements and allow us to analyse if movements are random (e.g. due to restlessness) or repetitive (e.g. due to cycling movement of the leg.) *Physically active* is defined as walking, climbing stairs, sit-to-stand, standing or other exercise in the upright position. In-bed cycling with the trunk below 60° is also characterised as being physically active. *Physically sedentary* is any seated position with the upper body elevated more than 60° from supine and with movement registrations of the upper body [11]. *Physically inactive* is defined as a seated or lying position and no registrations of small movements during the day between 7 a.m. and 10 p.m. Finally, *sleeping* is defined as a lying physically inactive period between 10 p.m. and 7 a.m. At the end of the two seven-day periods, we will primarily aggregate data into the percentage of time for each category; only days without any significant missing data will be included. A significant amount of missing data is more than 1 h of missing data during the daytime (7 a.m - 10 p.m) or 1 h during the night-time (10 p.m. - 7 a.m).

*Potential factors that might influence the outcome:* Complications or infections can affect physical activity levels during hospitalisation. Therefore, data on complications or factors affecting physical activity are registered during P1 and P2, with yes/no, frequency and date if relevant. We will also register number of days admitted to the rehabilitation unit (Table 1 and Appendix 1).

*Functional outcomes at discharge:* EFA total score and sensory-motor subscale scores (items 9–15); FIM total score, FIM-Motor and FIM-Cog subscale scores; 30-s Sit-to-Stand test [28]; Functional Ambulation Category (FAC) [29]; 10-m walk test [30] and the 6-min walk test [31].

All data obtained from the digital medical record are entered into REDCap (Research Electronic Data Capture) hosted at Capital Region Denmark and established for this study [32,33]. REDCap is an online software for obtaining and storing research data for individual records. Activity data will be exported to the REDCap database as well. All data will be collected and handled by the research group.

### 2.3 Statistical analyses

The estimated sample size is based on the recommendations by Whitehead et al. for outcomes in pilot studies with an expected low effect size, a power of 90 % and an alpha level of 5 % [34]. Therefore, to reduce the risk of inflation of the upper confidence limits, 100 patients will be included. The secondary objectives are based on mixed-effects models. No prior studies are available to inform a sample size calculation.

We will present patient demographics and characteristics as mean values and standard deviation (SD) or medians with interquartile ranges for continuous or discrete data and as counts and percentages for dichotomous and nominal data. For each physical activity category, we will quantify the total amount of time in minutes (mean [SD] and median [range]) and present the proportions of the total measured time, including 95 % confidence intervals during P1 and P2. Furthermore, proportions will be graphically presented.

To investigate the amount of physical activity in the two periods, a mixed-effect linear regression will be used after control of statistical assumptions to analyse continuous variables, and mixed-effect logistic regression will be used after control of statistical assumptions to analyse dichotomous outcomes (physically active vs other categories). We will use either fixed-effect models or non-parametric tests to count outcomes not fulfilling the assumptions.

Using the percentage of time spent in each of the four activity levels as the outcome variable at P1 and P2 (dependent variable), we will use multiple linear regression analysis to investigate whether any of the descriptive variables (i.e., patient characteristics or other measurements) are associated with the level of physical activity. We will test for correlations, interactions, and variance in residuals to investigate whether the model assumptions are valid. Finally, FIM-motor and FIM-Cog subscales, the 30-s sit-to-stand test, and the Functional ambulation category will be analysed as outcome variables in two models using the physical activity level as a descriptive variable and adjusting for age, gender, FOUR score, primary diagnosis, and number of comorbidities. Both adjusted and unadjusted analyses will be presented.

This study uses a non-randomised method, so no conclusive causalities can be drawn regardless of the *P*-values. An exploratory alpha level in this study will be set at 0.05.

#### 2.3.1 Subgroups/interaction

We will divide the patients into subgroups according to their physical activity level during P1. Suppose a clear distinction can be made at the activity level. In that case, we will characterise patients as highly active (patients with the highest amount of time defined as physically active periods), sedentary active (patients mostly seated but detectable adjustments and movements), or inactive. If no clear distinction is possible, the activity level will be recorded as a continuous variable in minutes.

For the first subgroups, logistic regression will be made using the activity subgroups as the descriptive variable and functional outcome as the outcome variable. Age, gender, and primary diagnosis will be incorporated as adjusting variables.

#### 2.3.2 Missing data

Missing data are problematic for statistical analysis. The missing at-random assumptions are often hard to justify, and the interpretation and generalisations are difficult to establish [35]. The primary analysis will use complete case analysis, but missingness must be used to interpret the results. Therefore, if less than 5 % of the data are missing, we will only conduct a complete case analysis. If 5 %–25 % of data are missing (on the outcome and descriptive variables), we will consider performing a mixed-effects model imputing the missing data as sensitivity analyses. Suppose more than 25 % of data on a descriptive variable is missing. In that case, we will conduct a complete case analysis and a best-worst-case analysis of data on physical activity by grouping participants as either physically active or inactive as a sensitivity analysis. In any case, such analyses will be at high risk of bias [35].

Patients with agitated behaviour can sometimes be challenging to manage in the department. If we, during the study experience, that the physical activity monitors are problematic for managing agitation, we will discontinue the measurements. Data will then be missing.

#### 2.3.3 Bias

Non-randomised trials are prone to bias for several reasons [36]. Firstly, this study investigates physical activity using body-worn activity monitors when the application of the monitor itself may lead to patients or clinicians becoming more aware of the importance of physical activity. Therefore, bias could be introduced. Although the patient's clothes hide the monitor from the clinical staff due to the placement on the femur and torso, we cannot rule this out.

Secondly, social activity may also affect physical activity if relatives or friends can motivate the patient to move more. Monitoring visits or social interactions are not possible in this study, but any activity outside the daytime (after 5 p.m.) is registered and discussed for potential bias.

Thirdly, patients with severe acquired brain injury often present with comorbidities and complications that may reduce physical activity. Several variables affecting physical activity levels are collected to better understand physical activity patterns.

## 3 Discussion

This protocol describes an observational study performed at a rehabilitation department for patients with moderate to severe acquired brain injuries. Physical inactivity has been associated with several lifestyle-related diseases and, in patients with neurological diseases, with poorer physical outcomes and reduced survival [37]. Studies in animals have shown that increased physical activity (primarily voluntarily) results in an increased level of the brain-derived neurotrophic factor. This protein has neuroprotective abilities related to increased cortical plasticity [38,39]. Therefore, facilitating physical activity in patients with acquired brain injury may improve physical or cognitive function [40].

Physical activity monitors may also inform trials investigating physical interventions on the amount of exercise performed in the intervention and the control group besides the compared interventions. This information can be crucial when measuring the effect of physical rehabilitation interventions and increase the chance of succeeding in producing future meaningful evidence. Data from this trial can be used to inform randomised clinical trials on which physical activity level could be expected in a standard care control group and inform the estimation of participants in such a trial. Furthermore, simple interventions focusing on increasing physical activity could be relevant to many neurological diagnoses. The hypothesis in this study is that we can predict who is at risk of being physically inactive, which can also prove to be an essential tool for future rehabilitation.

There are some limitations to this study. The design is an observational study, which is prone to bias. On the other hand, we have no access to the collected data on the physical activity level before the last patient is included. Therefore, knowledge of physical activity level will not directly affect data collection on the independent variables used in the analysis.

The study may also be at risk of losing data if the adhesiveness of the monitors is reduced over time, and they consequently fall off. We attempt to prevent this through daily assessments by the clinical staff, with instructions to change the adhesive patches if they are loosely attached.

## 4 Conclusions

This study will describe physical activity in four categories of activity patterns in patients admitted to in-hospital rehabilitation after moderate to severe acquired brain injury. Physical activity patterns in neurorehabilitation services might be highlighted and help inform and educate rehabilitation staff.

## Author contribution statement

Vibeke Wagner: Conceived and designed the experiments; Wrote the paper; Pi Gravesen: Conceived and designed the experiments; Wrote the paper; Emma Ghaziani: Conceived and designed the experiments; Wrote the paper; Markus Harboe Olsen: Conceived and designed the experiments; Wrote the paper; Christian Gunge Riberholt: Conceived and designed the experiments; Wrote the paper.

## Funding statement

The operational expenses are fully covered by the Department of Neurorehabilitation, Division of Brain Injury at Rigshospitalet. During the project, we will apply for external funding. A grant from the 10.13039/501100003554Lundbeck Foundation for healthcare research funds CGR's salary.

## Financial disclosure

None reported.

## Data availability

After this study is completed and the results published, all collected data will be anonymised and uploaded to the Zenodo repository (www.zenodo.org).

## Additional information

The study does not require ethical approval by the Danish National Committee on Health Research Ethics (Journal-number: H-22031667; 08.16.2022). The study is registered on clinicaltrials.gov (NCT05571462; 09.29.2022) before the inclusion of the first participant. The first study participant was enrolled 4th of November 2022.

## Declaration of competing interest

The authors declare that they have no known competing financial interests or personal relationships that could have appeared to influence the work reported in this paper.

## References

[1] Okely A.D., Kontsevaya A., Ng J., Abdeta C. (2021). WHO guidelines on physical activity and sedentary behavior. Sports Medicine and Health Science.

[2] Norvang O.P., Hokstad A., Taraldsen K., Tan X., Lydersen S., Indredavik B. (2018). Time spent lying, sitting, and upright during hospitalization after stroke: a prospective observation study. BMC Neurol..

[3] Adey-Wakeling Z., Jolliffe L., Eos Boccther, Hunter P., Morarty J., Cameron I.D. (2021). Activity, participation, and goal awareness after acquired brain injury: a prospective observational study of inpatient rehabilitation. Ann Rehabil Med.

[4] Kjeldsen S.S., Brodal L., Brunner I. (2020). Activity and rest in patients with severe acquired brain injury: an observational study. Disabil. Rehabil..

[5] Hassett L., Wong S., Sheaves E., Daher M., Grady A., Egan C. (2018). Time use and physical activity in a specialised brain injury rehabilitation unit: an observational study. Brain Inj..

[6] Bernhardt J., Chitravas N., Meslo I.L., Thrift A.G., Indredavik B. (2008). Not all stroke units are the same: a comparison of physical activity patterns in Melbourne, Australia, and Trondheim, Norway. Stroke.

[7] Teasell R., Dittmer D.K. (1993 Jun). Complications of immobilization and bed rest. Part 2: other complications. Can. Fam. Physician.

[8] Dittmer D.K., Teasell R. (1993 Jun). Complications of immobilization and bed rest. Part 1: musculoskeletal and cardiovascular complications. Can. Fam. Physician.

[9] Zeiler S.R., Krakauer J.W. (2013 Dec). The interaction between training and plasticity in the poststroke brain. Curr. Opin. Neurol..

[10] Bo Nielsen J., Willerslev-Olsen M., Christiansen L., Lundbye-Jensen J., Lorentzen J. (2015 Jan). Science-based neurorehabilitation: recommendations for neurorehabilitation from basic science. J. Mot. Behav..

[11] Riberholt C.G., Wagner V., Lindschou J., Gluud C., Mehlsen J., Møller K. (2020).

[12] Newman M., Barker K. (2012 Dec). The effect of supported standing in adults with upper motor neurone disorders: a systematic review. Clin. Rehabil..

[13] World Health Organization (2001).

[14] Ferguson T., Hons B., Phd C., Blake H., Hons B., Crozier A.J. (2022). Effectiveness of wearable activity trackers to increase physical activity and improve health: a systematic review of systematic reviews and meta-analyses. Rev Lancet Digit Heal.

[15] Larsen R.T., Wagner V., Korfitsen C.B., Keller C., Juhl C.B., Langberg H. (2022). Effectiveness of physical activity monitors in adults: systematic review and meta-analysis. BMJ.

[16] SENS motion®. Discrete Wearable Activity Sensor for Healthcare and Research [Internet]. [cited 2022 Nov 28]. Available from: https://sens.dk/en/.

[17] Pedersen B.S., Kristensen M.T., Josefsen C.O., Lykkegaard K.L., Jønsson L.R., Pedersen M.M. (2022 May 16). Validation of two activity monitors in slow and fast walking hospitalized patients. Rehabil Res Pract.

[18] Sundhedsstyrelsen © (2011).

[19] Sundhedsstyrelsen © (2014).

[20] Kleim J.A., Jones T.A. (2008). Principles of experience-dependent neural plasticity: implications for rehabilitation after brain damage. J. Speech Lang. Hear. Res..

[21] Von Elm E., Altman D.G., Egger M., Pocock S.J., Gøtzsche P.C., Vandenbrouckef J.P. (2007). The strengthening the reporting of observational studies in epidemiology (STROBE) statement: guidelines for reporting observational studies. Bull. World Health Organ..

[22] Vandenbroucke J.P., Von Elm E., Altman D.G., Gøtzsche P.C., Mulrow C.D., Pocock S.J. (2007). Strengthening the reporting of observational studies in epidemiology (STROBE): explanation and elaboration. PLoS Med..

[23] Bartholdy C., Gudbergsen H., Bliddal H., Kjærgaard M., Lykkegaard K.L., Henriksen M. (2018 Mar). Reliability and construct validity of the SENS Motion® activity measurement system as a tool to detect sedentary behaviour in patients with knee osteoarthritis. Arthritis.

[24] Wijdicks E.F.M., Bamlet W.R., Maramattom B.V., Manno E.M., McClelland R.L. (2005). Validation of a new coma scale: the FOUR score. Ann. Neurol..

[25] Hankemeier A., Rollnik J.D. (2015 Oct). The Early Functional Abilities (EFA) scale to assess neurological and neurosurgical early rehabilitation patients. BMC Neurol..

[26] Linacre J.M., Heinemann A.W., Wright B.D., Granger C.V., Hamilton B.B. (1994). The structure and stability of the functional independence measure. Arch. Phys. Med. Rehabil..

[27] Giacino J.T., Kalmar K., Whyte J. (2004). The JFK coma Recovery scale-revised: measurement characteristics and diagnostic utility. Arch. Phys. Med. Rehabil..

[28] Johansen K.L., Stistrup R.D., Schjøtt C.S., Madsen J., Vinther A. (2016 Oct). Absolute and relative reliability of the timed ‘up & go’ test and ‘30second chair-stand’ test in hospitalised patients with stroke. PLoS One.

[29] Mehrholz J., Wagner K., Rutte K., Meißner D., Pohl M. (2007 Oct). Predictive validity and responsiveness of the functional ambulation category in hemiparetic patients after stroke. Arch. Phys. Med. Rehabil..

[30] Busk H., Holm P., Skou S., Seitner S., Siemsen T., Wienecke T. (2022 Feb). Inter-rater reliability and agreement of 6 Minute Walk Test and 10 Meter Walk Test at comfortable walk speed in patients with acute stroke. Physiother. Theory Pract..

[31] Macchiavelli A., Giffone A., Ferrarello F., Paci M. (2021). Reliability of the six-minute walk test in individuals with stroke: systematic review and meta-analysis. Neurol. Sci..

[32] Harris P.A., Taylor R., Thielke R., Payne J., Gonzalez N., Conde J.G. (2009 Apr). Research electronic data capture (REDCap)-A metadata-driven methodology and workflow process for providing translational research informatics support. J. Biomed. Inf..

[33] Harris P.A., Taylor R., Minor B.L., Elliott V., Fernandez M., O'Neal L. (2019 Jul 1). The REDCap consortium: building an international community of software platform partners. J. Biomed. Inf..

[34] Whitehead A.L., Julious S.A., Cooper C.L., Campbell M.J. (2016 Jun). Estimating the sample size for a pilot randomised trial to minimise the overall trial sample size for the external pilot and main trial for a continuous outcome variable. Stat. Methods Med. Res..

[35] Jakobsen J.C., Gluud C., Wetterslev J., Winkel P. (2017). When and how should multiple imputation be used for handling missing data in randomised clinical trials - a practical guide with flowcharts. BMC Med. Res. Methodol..

[36] Savović J., Jones H., Altman D., Harris R., Jűni P., Pildal J. (2012 Sep). Influence of reported study design characteristics on intervention effect estimates from randomised controlled trials: combined analysis of meta-epidemiological studies. Health Technol. Assess..

[37] Jørgensen H.S., Nakayama H., Raaschou H.O., Olsen T.S. (1994). Stroke in patients with diabetes. The Copenhagen Stroke study. Stroke.

[38] Ploughman M., Granter-Button S., Chernenko G., Attwood Z., Tucker B.A., Mearow K.M. (2007). Exercise intensity influences the temporal profile of growth factors involved in neuronal plasticity following focal ischemia. Brain Res..

[39] Kramer S.F., Hung S.H., Brodtmann A. (2019). The impact of physical activity before and after stroke on stroke risk and Recovery: a narrative review. Curr. Neurol. Neurosci. Rep..

[40] Fini N.A., Bernhardt J., Said C.M., Billinger S.A. (2021). How to address physical activity participation after stroke in research and clinical practice. Stroke.

