# Supplementary material for: Mapping physical activity patterns in hospitalised patients with moderate to severe acquired brain injury - MAP-ABI: Protocol for an observational study
Source: Heliyon. 2023 Nov 8;9(11):e21927. doi: 10.1016/j.heliyon.2023.e21927 (PMC10682202; doi:10.1016/j.heliyon.2023.e21927)
Supplement: Multimedia component 1 [file mmc1.docx]

**Appendix 1**

**Mapping physical activity patterns in hospitalised patients with moderate to severe acquired brain injury - MAP-ABI: protocol for an observational study.**

Vibeke Wagner^1^, Pi Gravesen^1^, Emma Ghaziani^1^, Markus Harboe Olsen^2^, Christian Gunge Riberholt^1^

^1^Department of Brain and Spinal Cord Injury, The Neuroscience Centre, Copenhagen University Hospital – Rigshospitalet, Copenhagen, Denmark. ^2^Department of Neuroanaesthesiology, The Neuroscience Centre, Copenhagen University Hospital – Rigshospitalet, Copenhagen, Denmark

**Definition of variables – complications.**

Infections: The number of infections treated with antibiotics will be registered.

Observed syncope: A syncope is defined by reduced blood pressure or/and increased heart rate to a degree where the patient looses consciousness.

Visual deficits: Defined as neglect, hemianopsia, visual processing problems, or eye-movement problems. A specialised neurologist in the department must confirm deficits.

Nausea and dizziness: Nausea and dizziness are subjective symptoms. Nausea and dizziness are present if the patient has a relevant complaint (e.g. describes dizziness as rotatory or nautical) or with explicit vomiting.
